# COVID‐19‐Associated Cytotoxic Lesions of the Corpus Callosum in Chinese Patients: A Retrospective Study

**DOI:** 10.1002/brb3.70547

**Published:** 2025-05-28

**Authors:** Chenyi Wan, Menghua Li, Yanyan Yu, Si Luo, Daojun Hong, Meihong Zhou, Yu Zhu

**Affiliations:** ^1^ Department of Neurology, The First Affiliated Hospital, Jiangxi Medical College Nanchang University Nanchang China; ^2^ Rare Disease Center, the First Affiliated Hospital, Jiangxi Medical College Nanchang University Nanchang China; ^3^ Institute of Neurology, Jiangxi Academy of Clinical Medical Science, The First Affiliated Hospital, Jiangxi Medical College Nanchang University Nanchang China; ^4^ Key Laboratory of Rare Neurological Diseases of Jiangxi Provincial Health Commission, Jiangxi Medical College Nanchang University Nanchang China

**Keywords:** corpus callosum, neuroimaging, inflammatory, encephalopathy, SARS‐CoV‐2

## Abstract

**Background and purpose:**

Cytotoxic lesions of the corpus callosum (CLOCCs) are a rare clinicoradiologic syndrome, exceptionally rare in association with coronavirus disease (COVID‐2019). This study aimed to investigate the neurological features of COVID‐19‐associated CLOCCs and gain insights into their underlying pathophysiology.

**Methods:**

A retrospective evaluation was conducted on patients with COVID‐19‐associated CLOCCs admitted to our neurological diseases unit. The evaluation included comprehensive analysis of clinical presentations, laboratory findings, and radiological data.

**Results:**

From December 17, 2022, to December 31, 2023, our center identified CLOCCs in eight individuals with clinically established COVID‐19 who underwent brain magnetic resonance imaging (MRI). The majority of patients (7/8) presented with fever preceding neurological symptoms. The spectrum of neurological findings encompassed altered consciousness (5/8), headache (4/8), cognitive and behavioral disturbances (4/8), ataxia (2/8), dysarthria (2/8), pyramidal signs (2/8), and visual impairments (2/8). Peripheral blood markers of inflammation and cytolysis revealed trends that paralleled disease progression. Elevated cerebrospinal fluid protein levels were observed in a single patient, whereas cell counts, glucose, and chloride levels were unremarkable. Treatment with glucocorticoids and antiviral medications led to complete clinical remission, with subsequent MRIs (7/8) showing radiological improvements within 3 days to 2 weeks posttreatment.

**Conclusions:**

Our study shows that CLOCCs associated with COVID‐19 are characterized by a favorable prognosis and distinct MRI features, similar to those observed in other clinical contexts. This underscores the importance of including CLOCCs in the differential diagnosis of COVID‐19 and highlights the need for ongoing research to address the neurological condition of SARS‐CoV‐2 infections and to inform preventive and therapeutic strategies.

## Graphical abstract

We report the clinical characteristics, radiological features, and changes in inflammatory markers throughout the entire course of illness in eight patients with COVID‐19‐associated CLOCCs. The neurological symptoms or signs in these patients included altered consciousness, headache, cognitive impairment, ataxia, dysarthria, pyramidal signs, and visual disturbances. Additionally, monitoring the dynamic changes in peripheral blood inflammatory markers was found to be helpful in reflecting the prognosis of the disease.

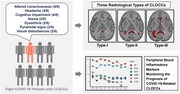

## 1 Introduction

Coronavirus disease 2019 (COVID‐19), caused by severe acute respiratory syndrome coronavirus 2 (SARS‐CoV‐2), has been associated with a spectrum of neurological complications (Almqvist et al. 2020), collectively termed NeuroCOVID‐19 (Fehr and Perlman 2015). Acute NeuroCOVID‐19 has been reported to manifest as epilepsy, anosmia, headache, nausea, and delirium (Yum and Shin 2022). These complications range from mild symptoms such as anosmia and headache to more severe manifestations like encephalopathy and stroke. Among these, cytotoxic lesions of the corpus callosum (CLOCCs) have emerged as a distinct radiological pattern in adolescents and adults with NeuroCOVID‐19 (LaRovere et al. 2021).

A recent report identified CLOCCs‐like findings on brain magnetic resonance imaging (MRI) in three out of 73 COVID‐19 patients (Chougar et al. 2020). However, the prevalence of COVID‐19‐associated CLOCCs may be underestimated as not all patients undergo MRI examinations. During the acute phase of CLOCCs, localized increases in signal intensity are observed on diffusion‐weighted image (DWI) sequences, along with a decrease in signal on apparent diffusion coefficient (ADC), indicating cytotoxic edema (Maeda et al. 2006; Starkey et al. 2017). These lesions, previously referred to by various names including mild encephalitis/encephalopathy with reversible splenial (MERS) lesions and reversible splenial lesion syndrome (RESLES), are now collectively recognized as CLOCCs. (Tetsuka 2019). The pathophysiological mechanism underlying CLOCCs involves inflammatory processes triggered by cytokines like Interleukin‐6 (IL‐6), leading to glutamate accumulation and astroglial inflammation. Additionally, an imbalance in arginine pressor (AVP)/antidiuretic hormone (ADH) levels can induce hyponatremia, causing intracellular water retention and cytotoxic edema (Blaauw and Meiners 2020; Saito et al. 2022).

While CLOCCs have been reported in the context of various diseases, their association with COVID‐19 is exceptionally rare. A recent study identified CLOCCs‐like findings in a small proportion of COVID‐19 patients (Loh et al. 2005). However, the exact mechanisms by which SARS‐CoV‐2 may cause neurological manifestations, including direct neuropathic effects, AVP/ADH‐induced hyponatremia, or excessive inflammatory responses triggered by cytokine release from the host immune system, remain to be determined.

In this retrospective study, we aimed to investigate the neurological features of COVID‐19‐associated CLOCCs and gain insights into their underlying pathophysiology, emphasizing the importance of including CLOCCs in the differential diagnosis of future SARS‐CoV‐2 variant outbreaks.

## 2 Patients and Methods

From December 2022 to January 2023, eight patients with COVID‐19‐related CLOCCs were diagnosed at the First Affiliated Hospital of Nanchang University. The inclusion criteria were as follows: (1) patients confirmed to have SARS‐CoV‐2 infection, with positive result of PCR or antigen testing; (2) presence of COVID‐19 symptoms within 3 weeks prior to onset; (3) brain MRI showing restricted diffusion on corpus callosum DWI and lower signal intensity on ADC maps, with mild hyperintensity on T2‐weighted imaging (T2WI); and (4) hospitalized patients with detailed clinical data records. The exclusion criteria included (1) pre‐existing evidence of corpus callosum lesions and (2) exclusion of other causes of corpus callosum abnormalities (such as infections related to non‐COVID‐19, acute trauma, pregnancy, epilepsy or antiepileptic drugs, chemotherapy, and alcohol intoxication). All procedures were approved by the Ethics Committee of the First Affiliated Hospital of Nanchang University.

### 2.1 Clinical Characteristics and Laboratory Parameters

All patients underwent rigorous clinical evaluations by at least two neurologists aiming to exclude other causes of neurological injury, such as alcohol or drug intoxication, hypoxemia, thiamine or vitamin B12 deficiency, epilepsy, metabolic encephalopathy, and non‐NeuroCOVID‐19 disorders. Demographic characteristics and clinical data including age, gender, medical history, onset symptoms, COVID‐19 symptoms during hospitalization, neurological symptoms, respiratory function assessment, MRI features, treatment strategies, and outcomes of neurological disorders were recorded. Laboratory tests were conducted to identify the presence of CLOCCs, including peripheral blood cell count, pro‐inflammatory molecules (IL‐6 and C‐reactive protein), inflammatory cytokines, cellular lysis marker (e.g., lactate dehydrogenase), and coagulation function. Thyroid hormones, creatine kinase, and electrolytes were also measured. All peripheral blood samples were collected within 6 h after admission. All patients underwent a 3‐T MRI scan, including T1‐weighted, T2‐weighted, three‐dimensional fluid‐attenuated inversion recovery (FLAIR, both with and without gadolinium), DWI, and ADC maps. All MRI images were carefully interpreted by at least one radiologist and one neurologist.

### 2.2 Statistical Analyses

All statistical analyses and visualizations were performed by using the R software (version 4.2.2). All laboratory variables were described as means ± SD. Continuous variables were compared using nonparametric Wilcoxon tests with an irregular distribution or Student's *t*‐tests with a normal distribution.

## 3 Results

### 3.1 Clinical Evaluation

**FIGURE 1:**
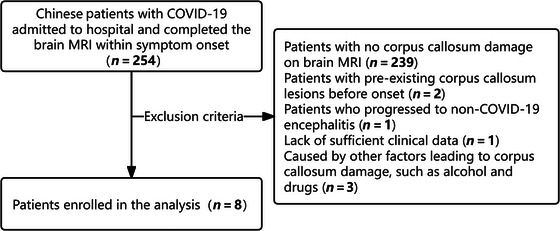
Selection of study participants.

Between December 17, 2022, and December 31, 2023, a total of 5679 patients were hospitalized for COVID‐19 infection, and 254 patients underwent brain MRI examinations, with an average age of 64.42 years (range: 15–96 years). Exclusions were due to pre‐existing corpus callosum lesions in two patients, non‐COVID‐19 encephalitis in one, insufficient clinical data in one, and other factors like alcohol and antiepileptic drugs causing corpus callosum damage in three patients (Figure 1). Eight patients (four males and four females) were diagnosed with CLOCCs at the Neurology Center of the First Affiliated Hospital of Nanchang University. The average age was 31.13 years (range: 15–82 years), with more than half of the patients being younger than 20 years old. None of the patients had a history of neurological disorders or pre‐existing neurological symptoms or signs. Table 1 summarizes the general characteristics, clinical symptoms, radiological findings, laboratory test results, and treatment strategies of these eight patients. Severe COVID‐19‐related rhabdomyolysis was present in both Cases 6 and 7. The neurological symptoms included altered consciousness (5/8), headache (4/8), psychiatric abnormalities (4/8), ataxia (2/8), dysarthria (2/8), pyramidal tract signs (2/8), and visual disturbances (2/8).

**TABLE 1:** Characteristics of eight patients with cytotoxic lesions of the corpus callosum after severe acute respiratory syndrome coronavirus 2 (SARS‐CoV‐2) infection.

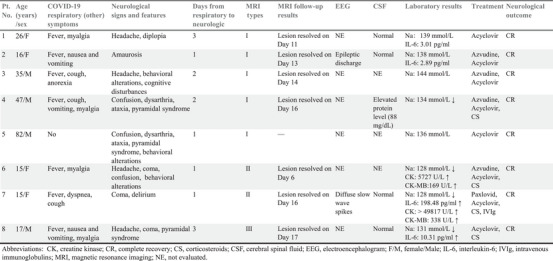

**FIGURE 2:**
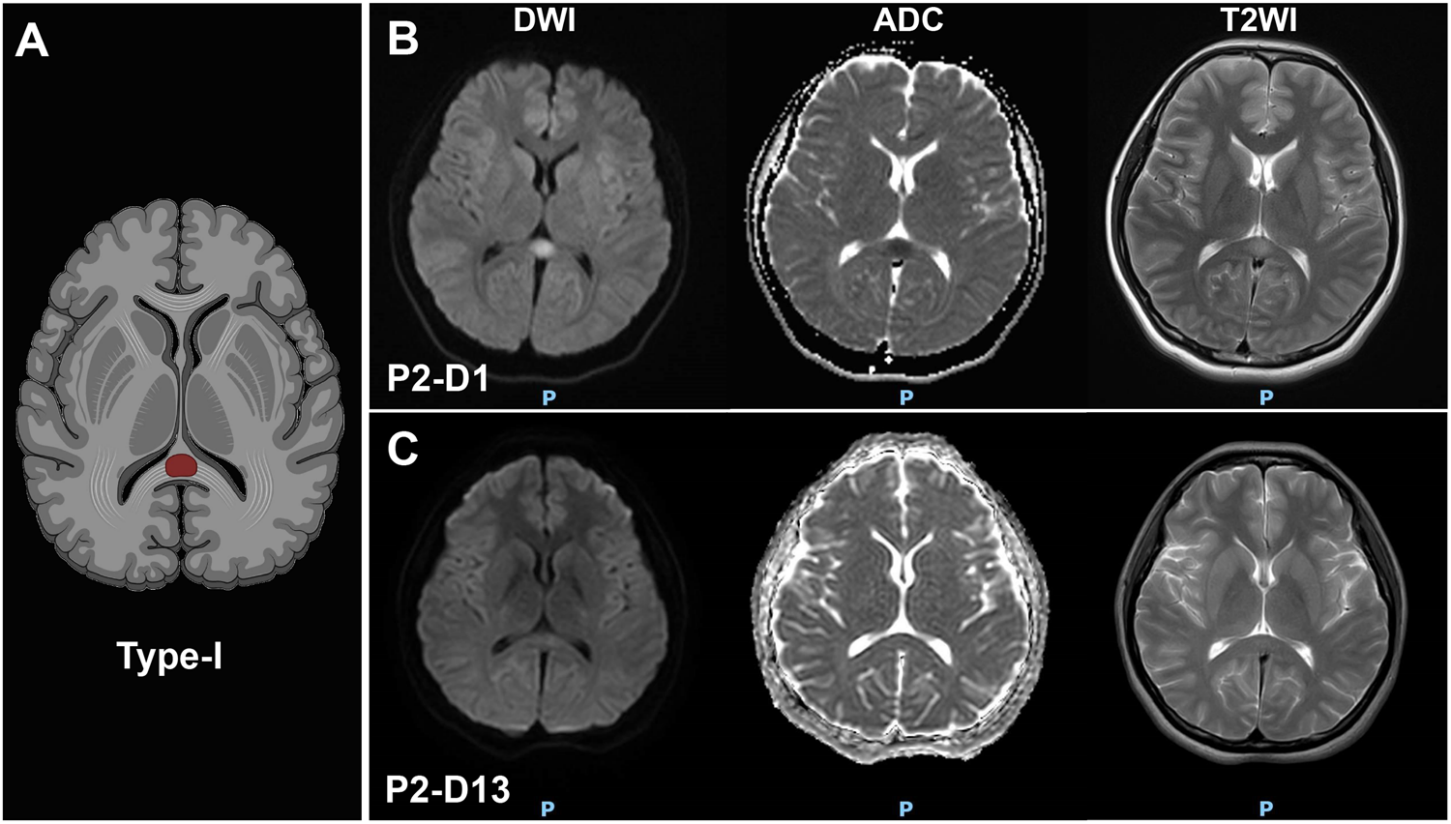
The Type‐I of MRI‐defined CLOCCs in COVID‐19 patients. Schematic diagram of Type‐I (A). Case 2 demonstrated the first pattern of CLOCCs, with an ovoid area of restricted diffusion in the central part of the splenium observed on MRI on the day of symptom onset (B). which resolved by the 13th day when symptoms improved (C).

**FIGURE 3:**
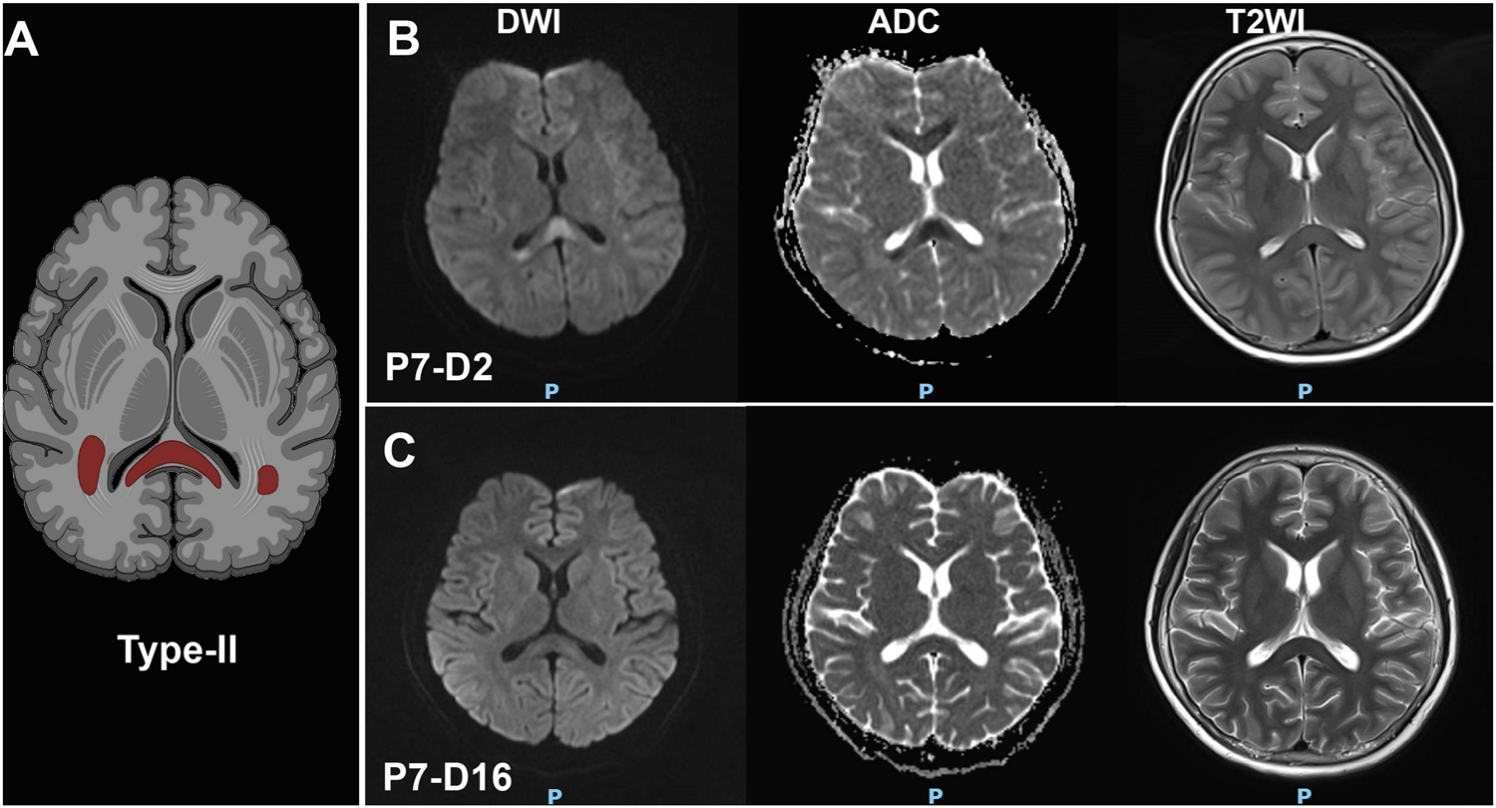
The Type‐II of MRI‐defined CLOCCs in COVID‐19 Patients. Schematic diagram of Type‐II (A). In Case 7, lesions involving the splenium and bilateral corpus callosum fibers were present at onset (B), with substantial resolution noted by the 16th day (C).

**FIGURE 4:**
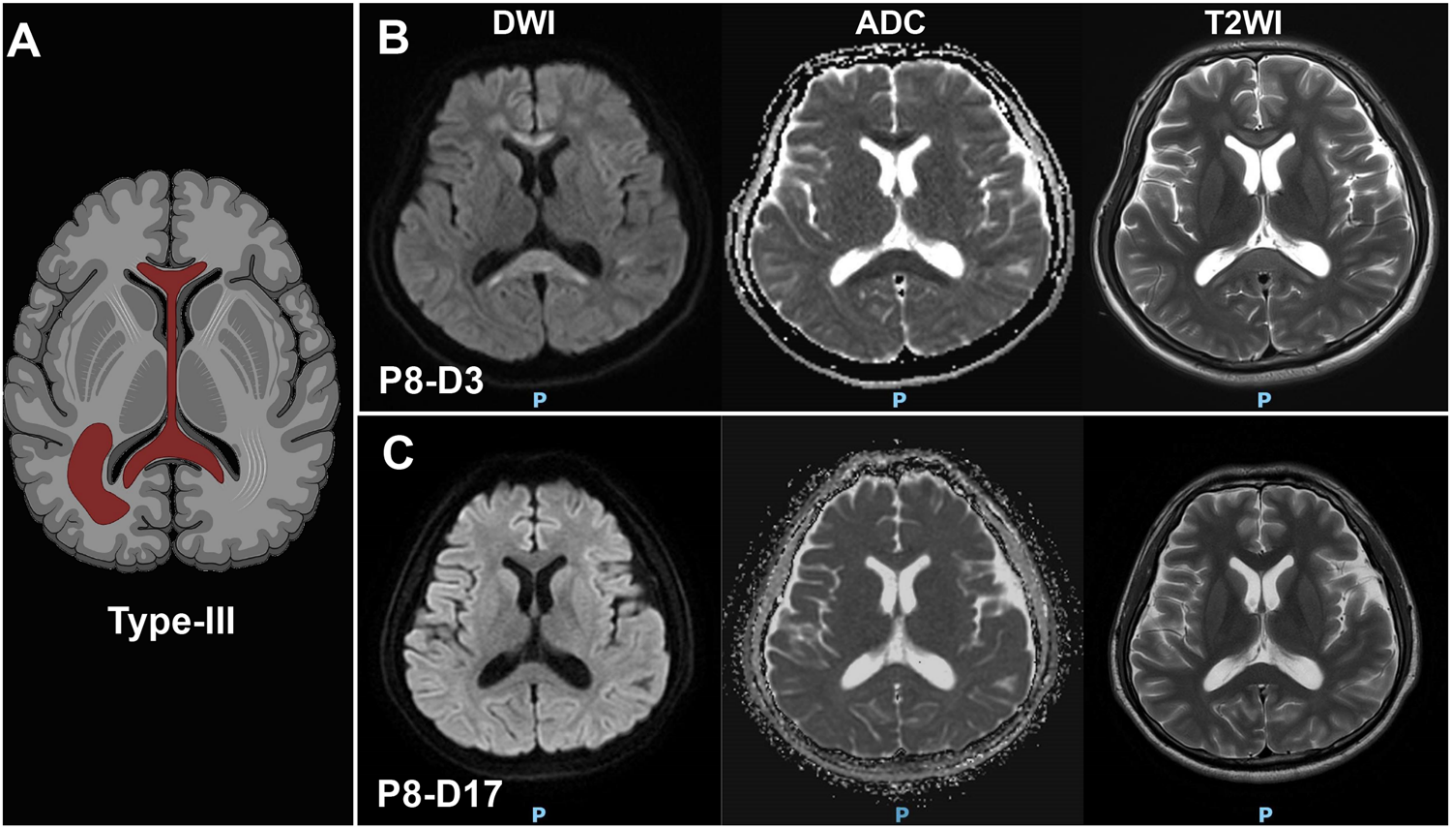
The Type‐III of MRI‐defined cytotoxic lesions of the corpus callosum (CLOCCs) in COVID‐19 patients. Schematic diagram of Type‐III (A). Case 8 exhibited diffuse involvement of the corpus callosum and periventricular white matter (B), with resolution upon clinical improvement (C).

All patients developed neurological symptoms within 3 days of COVID‐19 symptom onset, and the corpus callosum damage in those who underwent follow‐up brain MRI examinations was resolved within 2 or 3 weeks. Three types of CLOCCs were observed: Type‐I (Figure 2): small circular or elliptical lesions located centrally in the splenium (Cases 1–5). Type‐II (Figure 3): lesions extending laterally from the splenium along the corpus callosum fibers (Cases 6 and 7). Type‐III (Figure 4): lesions extending into the anterior part of the corpus callosum (Case 8). Cases 2 and 7 underwent electroencephalogram examinations, revealing epileptic discharges in Case 2 and diffuse slow wave spikes throughout the whole brain in Case 7.

### 3.2 Laboratory Results

**FIGURE 5:**
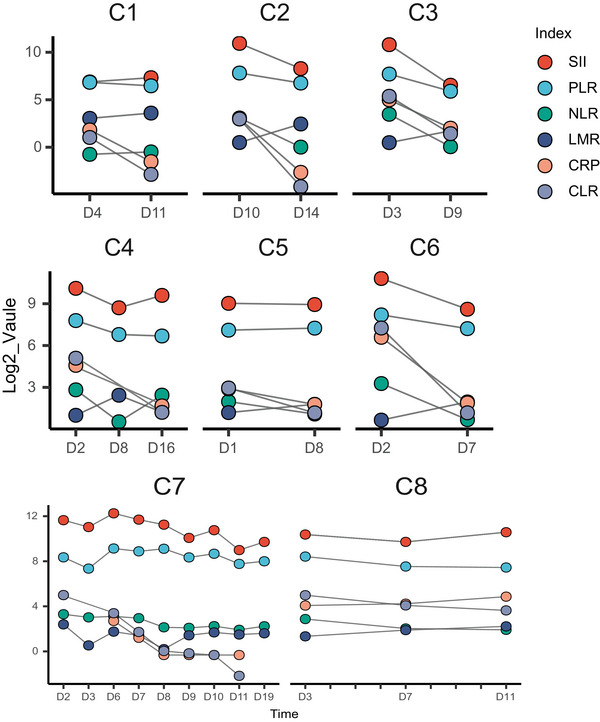
Temporal profile of inflammatory biomarkers reflecting disease evolution in CLOCCs. CLR, C‐reactive protein‐to‐lymphocyte ratio; CRP, C‐reactive protein; LDH, lactate dehydrogenase; LMR, lymphocyte‐to‐monocyte ratio; NLR, neutrophil‐to‐lymphocyte ratio; PLR, platelet‐to‐lymphocyte ratio; SII, systemic immune‐inflammation index.

**FIGURE 6:**
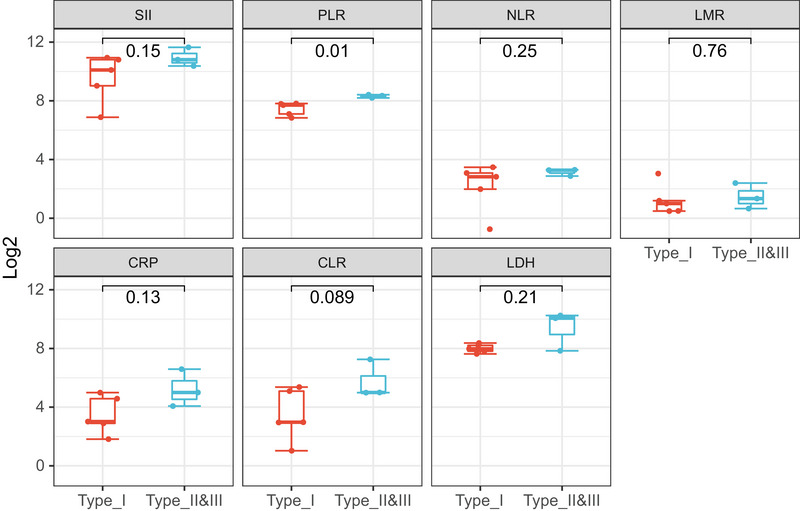
Comparison of Laboratory Indicators between Type‐I and Type‐II and ‐III Patients. CLR, C‐reactive protein‐to‐lymphocyte ratio; CRP, C‐reactive protein; LMR, lymphocyte‐to‐monocyte ratio; NLR, neutrophil‐to‐lymphocyte ratio; PLR, platelet‐to‐lymphocyte ratio; SII, systemic immune‐inflammation index. Student's *t*‐tests.

The laboratory analysis indicates that certain serum inflammatory markers (Figure 5) neutrophil‐to‐lymphocyte ratio (NLR: 7/8), platelet‐to‐lymphocyte ratio (PLR: 7/8), systemic immune‐inflammation index (SII: 7/8), C‐reactive protein (CRP: 7/8), CRP‐to‐lymphocyte ratio (CLR: 8/8), and IL‐6 (2/4) were elevated at the onset of the disease and gradually decreased as the disease resolved. Additionally, other markers lymphocyte‐to‐monocyte ratio (LMR: 6/8) were decreased in the early stages of the disease and progressively increased as the disease improved. It is worth noting that as all patients' corpus callosum lesions improved, the levels of CLR subsequently showed a continuous decline. The cytolysis index LDH showed a significant increase at the onset of the disease, followed by a gradual decrease. Furthermore, it was observed that half of patients had low levels of blood sodium, with four cases showing hyponatremia (normal range: 137–147 mmol/L). In comparison to Type‐I patients, Type‐II and Type‐III patients had higher serum inflammatory indices, including PLR, SII, CRP, CLR, and IL‐6, at the onset of the disease (Figure 6). Additionally, LDH levels were higher, and Type‐II and Type‐III patients had hyponatremia. It is worth noting that all eight selected patients underwent thyroid function tests. Three patients exhibited a mild elevation in thyroid‐stimulating hormone levels, accompanied by normal free triiodothyronine (T3) and thyroxine (T4) levels, indicative of subclinical hypothyroidism. To acquire the initial cerebrospinal fluid (CSF) profiles, lumbar punctures were performed on five patients. In each instance (5/5 cases), the CSF pressure, cell count, glucose, and chloride concentrations were within the normal range. However, an elevated total protein level was noted exclusively in the CSF sample from Patient 4. All patients tested positive for SARS‐CoV‐2 virus via RT‐PCR.

## 4 Discussion

CLOCCs, characterized by transient and reversible lesions involving the corpus callosum and potentially the adjacent white matter, have been observed in various conditions (DE Oliveira et al. 2020). It is worth noting that viral infections are becoming an increasingly common cause of CLOCCs (Arıkan et al. 2022; Mračková et al. 2020). Mechanisms that may make the corpus callosum susceptible to damage include structural features and blood supply characteristics. These factors may include adrenergic receptor deficiency (Starkey et al. 2017), impaired regulation of water and electrolyte homeostasis and ion transport (Sarigecili et al. 2021), excitatory amino acid toxicity (Anneken et al. 2008; Tetsuka 2019), oxidative stress injury (Reyes et al. 2023), and immune dysregulation‐mediated inflammatory infiltration (Yum and Shin 2022). It is important to consider these factors when assessing potential damage to the corpus callosum. SARS‐CoV‐2 has been found to impact the blood–brain barrier through various mechanisms, including those related to COVID‐19 (Abid et al. 2023).

COVID‐19‐related CLOCCs are often observed in adolescents and young adults, with more severe neurological impacts in these age groups, as seen in pediatric cases linked to multisystemic inflammatory syndromes (Davoodi et al. 2023; Elkhaled et al. 2020; Kubo et al. 2022; Ucan et al. 2022). Nevertheless, it is important to note that all patients experienced complete remission of neurological symptoms. A case series noted that 30 patients with COVID‐19‐associated CLOCCs exhibited neurological symptoms such as altered consciousness, ataxia, hallucinations, dysarthria, and psychotic disorders, aligning with literature reports (Kubo et al. 2022). Furthermore, neurological symptoms emerge rapidly following infection and then quickly resolve, suggesting that blood–brain barrier disruption leading to direct SARS‐CoV‐2 virus invasion or myelin damage is unlikely to be the direct cause of CLOCCs and is more likely secondary to internal environment imbalances. Additionally, clues from reports of CLOCCs even after SARS‐CoV‐2 vaccination suggest that direct viral injury may not be the primary cause of COVID‐19‐associated CLOCCs (Kubo et al. 2022).

The radiologic features of different types of CLOCCs were demonstrated by our eight patients, which were categorized into three types based on the extent of the corpus callosum lesions (Starkey et al. 2017). Type‐I lesions, localized to the splenium of the corpus callosum, were associated with more rapid recovery, while Type‐II and Type‐III, involving peripheral white matter or the anterior corpus callosum, were associated with more severe neurological symptoms. This suggests that the extent of corpus callosum involvement may influence the clinical presentation and recovery from CLOCCs. Regarding the indications for brain MRI in patients with COVID‐19, we suggest that brain MRI should be considered in patients presenting with acute neurological symptoms, particularly when these symptoms cannot be readily explained by other causes. Additionally, brain MRI may be warranted in cases where there is a suspicion of CLOCCs based on clinical presentation, as early identification can guide management and prognostication.

It is suggested that serologic inflammatory markers may serve as a better guide for disease assessment in patients. Inflammatory markers in our patients returned to normal levels upon resolution of neurological symptoms, suggesting that these markers, especially the CLR, may serve as useful indicators of disease activity. Higher levels of inflammatory markers and more dramatic inflammatory changes were observed in Type‐II and Type‐III patients. The observation of hyponatremia in some of our patients, particularly those with white lesions, supports the hypothesis that electrolyte imbalances may contribute to the pathogenesis of CLOCCs. Specifically, hypotonic hyponatremia has been linked to edema within the myelin sheaths, which can result in transient diffusion reduction on MRI (Takanashi et al. 2009). According to Takanashi et al. (2009), most of the MERS patients had mild hyponatremia, with a mean serum sodium level of 131.0 ± 4.1 mmol/L, which is also consistent with the results of the cases we observed.

Given the small number of patients with COVID‐19‐associated CLOCCs and the lack of established therapeutic guidelines, our treatment approach included a variety of antiviral and immunomodulatory therapies. The full recovery of all our patients without complications suggests that early and aggressive treatment may be beneficial, though larger studies are needed to establish best practices.

The retrospective nature of our study and the modest number of patients limit the generalizability of our findings. Although CLOCCs are relatively rare in COVID‐19 patients, they provide a specific imaging clue for the diagnosis of COVID‐19. While not pathognomonic, they boost diagnostic accuracy when combined with clinical and lab findings. Moreover, CLOCCs may reflect a broader inflammatory response, indicating COVID‐19 severity. Our observations highlight the importance of considering CLOCCs in the differential diagnosis of COVID‐19 patients presenting with neurological symptoms and underscore the need for further research to better understand the pathophysiology and optimal management of these lesions.

## 5 Conclusion

This study presents clinical and radiological findings from eight patients with COVID‐19‐associated CLOCCs, highlighting the favorable prognosis and the importance of considering CLOCCs in the differential diagnosis of neurological symptoms in COVID‐19 patients. The detection of inflammatory biomarkers in these patients suggests a potential role of inflammation in the pathogenesis of CLOCCs. Given the ongoing threat of SARS‐CoV‐2 variants, further research is needed to understand the full spectrum of COVID‐19's neurological impact and to guide future prevention and treatment strategies.

## Author Contributions

**Chenyi Wan**: conceptualization, data curation, writing – original draft, investigation. **Menghua Li**: data curation, investigation. **Yanyan Yu**: data curation. **Si Luo**: data curation. **Daojun Hong**: writing – review and editing, formal analysis. **Meihong Zhou**: formal analysis, writing – review and editing. **Yu Zhu**: conceptualization, formal analysis, writing – original draft.

## Conflicts of Interest

The authors declare no conflicts of interest.

### Peer Review

The peer review history for this article is available at https://publons.com/publon/10.1002/brb3.70547.

## Data Availability

All presented data in this study are available from the corresponding author upon reasonable request.
